# Live‐attenuated influenza vaccine effectiveness against hospitalization in children aged 2–6 years, the first three seasons of the childhood influenza vaccination program in England, 2013/14–2015/16

**DOI:** 10.1111/irv.12990

**Published:** 2022-05-09

**Authors:** Nicki L. Boddington, Punam Mangtani, Hongxin Zhao, Neville Q. Verlander, Joanna Ellis, Nick Andrews, Richard G. Pebody

**Affiliations:** ^1^ Immunisation and Vaccine Preventable Diseases Division UK Health Security Agency London UK; ^2^ London School of Hygiene and Tropical Medicine London UK; ^3^ WHO Regional Office for Europe World Health Organization Copenhagen Denmark

**Keywords:** children, influenza, influenza vaccine effectiveness, LAIV, screening method

## Abstract

**Introduction:**

In 2013, the United Kingdom began to roll‐out a universal annual influenza vaccination program for children. An important component of any new vaccination program is measuring its effectiveness. Live‐attenuated influenza vaccines (LAIVs) have since shown mixed results with vaccine effectiveness (VE) varying across seasons and countries elsewhere. This study aims to assess the effectiveness of influenza vaccination in children against severe disease during the first three seasons of the LAIV program in England.

**Methods:**

Using the screening method, LAIV vaccination coverage in children hospitalized with laboratory‐confirmed influenza infection was compared with vaccination coverage in 2–6‐year‐olds in the general population to estimate VE in 2013/14–2015/16.

**Results:**

The overall LAIV VE, adjusted for age group, week/month and geographical area, for all influenza types pooled over the three influenza seasons was 50.1% (95% confidence interval [CI] 31.2, 63.8). By age, there was evidence of protection against hospitalization from influenza vaccination in both the pre‐school (2–4‐year‐olds) (48.1%, 95% CI 27.2, 63.1) and school‐aged children (5–6‐year‐olds) (62.6%, 95% CI 2.6, 85.6) over the three seasons.

**Conclusion:**

LAIV vaccination in children provided moderate annual protection against laboratory‐confirmed influenza‐related hospitalization in England over the three influenza seasons. This study contributes further to the limited literature to date on influenza VE against severe disease in children.

## 1 INTRODUCTION

The United Kingdom previously had a long‐standing selective influenza vaccination program that targeted populations at higher risk of severe disease due to influenza. Historically, this approach had been targeted at those 65 years of age and over, and those less than 65 years of age in clinical risk groups, including pregnant women, and healthcare workers, and aimed to directly protect these groups.^1^ Despite the program, there was recognized to still remain a considerable burden of disease, both in the targeted groups, largely due to limited effectiveness of vaccination, and, prior to the COVID‐19 pandemic, no substantial increases in uptake for many years, as well as on‐going burden and transmission in the non‐targeted groups, such as children.^1^

It is estimated that between 10% and 30% of children are infected with influenza annually^2^, ^3^, ^4^ and, although most often influenza infection is self‐limiting, complications leading to hospitalization can occur, particularly in those under 5 years of age and in children with chronic medical conditions. In the United Kingdom, the youngest children have the highest influenza‐related admission rates of all ages.^5^, ^6^, ^7^ In addition, children are recognized to play a key role in the transmission of the influenza virus in the wider community.^8^, ^9^, ^10^

In 2012, the Joint Committee on Vaccination and Immunisation (JCVI) considered the evidence for extending influenza immunization to healthy children using the newly licensed live‐attenuated influenza vaccine (LAIV) and recommended universal annual vaccination of all children aged 2–16 years for influenza with LAIV in England.^11^ The program was introduced incrementally from the start of the 2013/2014 influenza season, during which initially children aged 2–3 years were targeted nationally through general practice, as well as primary school aged children in seven geographical pilot areas in England. Since then, the program has been rolled out incrementally by adding additional age cohorts each season.

An important component of any new vaccination program is measuring how effective it is. The direct effect of a vaccine can be assessed after introduction in the targeted population using observational vaccine effectiveness (VE) studies.^12^ LAIVs have been previously shown to provide good protection against influenza illness, although more recent observational studies have provided mixed results, with VE varying widely across seasons and other countries.^13^, ^14^, ^15^, ^16^ In particular, in the United States, studies demonstrated reduced protection of LAIV against influenza A/H1N1pdm09 infection in the 2013/14 and 2015/16 seasons.^15^, ^17^, ^18^ Studies from the United Kingdom, Finland and Canada have shown good overall effectiveness of LAIV in children, although effectiveness was generally lower, specifically for the influenza A/H1N1pdm09 component of the vaccine, compared with injected inactivated vaccine (IIV).^13^, ^14^, ^19^ This was despite a new component, A/Bolivia/559/2013 strain, being introduced to the vaccine in 2015/16. These findings resulted in a temporary suspension of the use of LAIV and greater reliance on IIVs in the United States in 2016/17 and 2017/18.^20^, ^21^, ^22^

Despite these findings, other studies conducted in England have shown maintained protection against more severe disease (i.e., hospitalization) due to influenza A/H1N1pdm09 in 2015/16.^23^, ^24^ Using the screening method, Pebody et al. estimated LAIV VE against laboratory‐confirmed hospitalization in 2–6‐year‐olds to be 48.3% when adjusted for age, geography, and month for influenza A/H1N1pdm09.^24^ Boddington et al. estimated LAIV VE against laboratory‐confirmed hospitalization due to influenza A/H1N1pdm09 in 2–16‐year‐olds to be 42.4% when adjusted for sex, risk‐group, age group, region, ethnicity, deprivation, and month of sample collection using the test‐negative design.^23^

This study aims to extend the findings of these studies by assessing the effectiveness of influenza vaccination in children during the first three seasons of the LAIV program and to assess if protection against hospitalization was maintained in the seasons prior to 2015/16 when the influenza A/California/7/2009(H1N1)pdm09 strain was used in the LAIV, and in 2015/16, when the new vaccine strain was introduced (A/Bolivia/559/2013).

Using three seasons gives a greater amount of data to provide more robust evidence for the continued rollout of the childhood influenza vaccination program in England. The study also provides an opportunity to assess the utility of the screening method in assessing VE against hospitalization; the findings for which might be relevant for other vaccination programs such as the COVID‐19 VE studies.

The objective of this study was to estimate VE of influenza vaccination in preventing hospital admissions of laboratory‐confirmed influenza infection in children aged 2–6 years during the 2013/14, 2014/15, and 2015/16 influenza seasons using the screening method.

## 2 METHODS

Using the screening method, vaccination coverage in children hospitalized with laboratory‐confirmed influenza infection was compared with vaccination coverage in children in the general population, adjusted for age group, week/month, and geographical area.

### 2.1 Details of the data collection

#### 2.1.1 Cases

Cases of severe influenza admitted to hospital were identified from the sentinel UK Severe Influenza Surveillance System (USISS): a national surveillance system that collects data on hospitalized laboratory‐confirmed influenza cases from a sentinel network of acute NHS hospital Trusts in England.^7^ A confirmed case was defined as an individual admitted to a USISS sentinel hospital with a laboratory‐confirmed influenza infection during the influenza surveillance periods of the 2013/14, 2014/15, and 2015/16 influenza seasons (i.e., between Week 40 and Week 20 of the respective seasons) that were of target age for vaccination during those seasons (described below). Hospital trusts were provided with testing criteria to ensure that all patients admitted to hospital with clinical signs or suspicion of influenza were tested for influenza.

For each case, data on age, sex, geography, date of onset of influenza‐like illness, date of hospitalization, and specific clinical risk group status were extracted from the USISS system. The target groups for vaccination that were included in the study were 2–3‐year‐olds in the 2013/14 season, 2–4‐year‐olds in the 2014/15 season and 2–6‐year‐olds in the 2015/16 season (Table 1).

**TABLE 1:** Target childhood vaccination groups in England, 2013–15, setting of vaccine delivery and method of vaccine uptake data collection

Target group	Vaccine delivery	Vaccine offered	Denominator	Vaccine uptake	Data available
Pre‐school children (aged 2, 3, and 4 years^a^	Via primary care (GPs)	LAIV^c^	Number of patients registered with GP (healthy + those with clinical risk factors)	Immform^d^	Cumulative weekly, monthly and end‐of‐season uptake data aggregated by CCG and presence/absence of clinical risk group
School‐aged children (school years 1 and 2, i.e., children aged 5 rising to 6 years and aged 6 rising to 7 years)^b^	Via schools (but some areas chose to vaccinate via pharmacies/GPs)	LAIV^c^	Total number of children eligible for influenza vaccination in the LA geography AND children educated out of school in the LA geography, using local education authority population figures (healthy + those with clinical risk factors)	Immform^d^ via a separate monthly reporting system	Cumulative monthly and end‐of‐season uptake data aggregated by local authority

Abbreviation: LAIV, live‐attenuated influenza vaccine.

^a^ Pre‐school children aged 2–3 years were vaccinated in 2013/14, and in 2014/15 and 2015/16, pre‐school children aged 2–4 years were vaccinated.

^b^ School‐aged children were included in the program from the 2015/16 season,

^c^ Unless medically contraindicated in which case IIV is offered, although this data was not captured for the school program.

^d^ Immform is the routine national vaccine uptake monitoring system, Influenza Immunisation Uptake Monitoring Programme.

#### 2.1.2 Vaccination uptake of cases

Vaccination history of cases was obtained by sending a standard data collection proforma by post to the cases' General Practitioner in England. The date of administration and whether the vaccine was administered by injection or intranasally were also collected. This information was used to determine the proportion of cases vaccinated (PCV). This data collection was limited to the seasons included in this study.

A case was considered vaccinated if they received at least one dose of influenza vaccine (LAIV) in the relevant influenza season more than 14 days before disease onset, as this was considered the minimum time period to achieve maximum protection. When onset date was missing, the sample date minus 2 days was used as a proxy (based on the median time among those cases in whom the information was available) and if sample date was unknown then the test date minus 3 days was used (again based on the median time among those cases in whom the information was available).

Cases vaccinated by injection (i.e., by IIV) were excluded since population vaccine uptake is not available by vaccine type. All of the children in the school aged cohort will have been offered LAIV; however, a small number of 2–4‐year‐olds may have received IIV if they were contraindicated to recieve LAIV (due to severe immunodeficiency, those receiving salicylate therapy, those who have active wheezing at the time of vaccination or severe asthma and some with egg allergy^1^).

Cases where the vaccination history was unknown, or they were vaccinated less than 14 days before onset of symptoms, were also excluded from the analysis. When the date of vaccination was missing, cases who were hospitalized after mid‐January in the respective seasons were assumed to be vaccinated at more than 14 days before onset, because the vast majority of vaccinations are completed by mid‐January. In addition, cases where the interval between onset of illness and swab date exceeded 7 days were also excluded due to well documented reduced test sensitivity for longer time periods between these two‐time points.^25^, ^26^, ^27^, ^28^

#### 2.1.3 Reference population vaccine uptake

Data on population vaccination coverage was obtained from ImmForm: the routine national vaccine uptake monitoring system in England.^29^

For the pre‐school ages, weekly data were extracted from ImmForm on the number of children registered in primary care and the number of children who received seasonal influenza vaccination during the study seasons. Data were available by age group, week, geographical area (CCG), and presence/absence of a clinical risk factor. Data were not available by vaccine type.

For vaccinations in school‐aged children, data were extracted from ImmForm on the number of children of school age and the number of school‐aged children who received seasonal influenza vaccination during the study seasons. Data were available by year group, geographical area (local authority [LA]) and month.

This information was used to determine the proportion of the population vaccinated (PPV).

### 2.2 Statistical analysis

Data were analyzed using Stata v. 13.1 (StataCorp., USA).

For the crude analysis, VE can be calculated as 1− the odds of vaccination in cases/odds of vaccination in the population, or as below:

$$\mathrm{VE}=1-\left(\mathrm{PCV}/\left(1-\mathrm{PCV}\right)\right)$$

$$\left(\mathrm{PPV}/\left(1-\mathrm{PPV}\right)\right)$$

where PPV is the proportion of the reference group vaccinated, and PCV is the proportion of the influenza cases vaccinated.

End‐of‐season vaccine uptake figures by target group were used for the crude PPV estimates. These crude estimates can be stratified according to the availability of vaccination coverage in the reference group such as by age group.

For the adjusted analysis, each case was matched to the appropriate PPV that best matched that case according to key confounding variables. For this analysis, PPV was available by age group/year group (2–4‐year‐olds, school years 1 and 2), geographical area (CCG/LA) and by week/month. To take into account the 14 days required to develop immunity, vaccine coverage data were offset by 14 days to provide an estimate of weekly effective influenza vaccine uptake.

The VE, controlling for age group, week/month, and geographical area, was then estimated using logistic regression with vaccination status of the case as the outcome variable, a constant fitted and the logit of the individually matched vaccine coverage as an offset according to the method of Farringdon.^30^

The presence/absence of risk group was collected on all hospitalized cases although population vaccine uptake by presence/absence of risk group was only available for the pre‐school age groups (2–4‐year‐olds). In a subgroup analysis for 2–4‐year‐olds, where risk factor information was available, PPV was assigned to cases that matched to each case by presence/absence of risk factor, as well as age group, geographical area, and week used above. Separately, because data completion on risk factor information was poor, a sensitivity analysis was also undertaken for the above group. In the first instance, all cases aged 2–4 years were assumed to have a risk factor, then second, they were all assumed not to have a risk factor. Stratified VE estimates were also estimated for those with/without a risk factor.

This work was undertaken as a routine public health function to monitor vaccination programs. Public Health England (PHE), now the UK Health Security Agency (UKHSA), holds permissions to collect data under Section 251 of the National Health Service Act 2006 and the 2002 Health Service (Control of Patient Information) regulations, as part of the monitoring of the performance of national vaccination programs.

## 3 RESULTS

### 3.1 Description of cases

**FIGURE 1:**
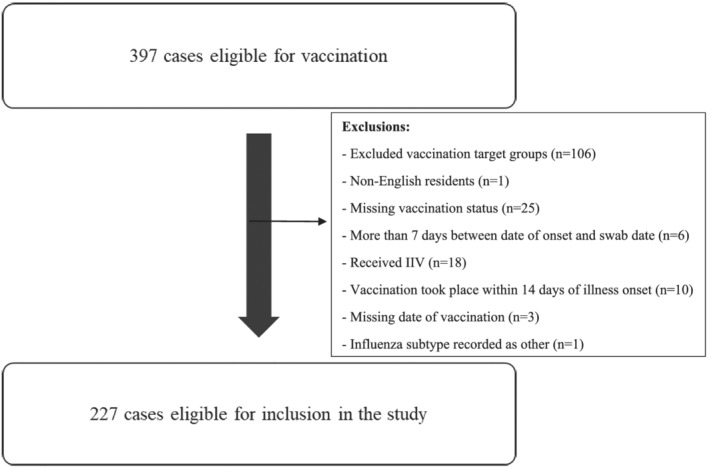
Case inclusion and exclusion flowchart

Three hundred and ninety‐seven cases eligible for vaccination were reported to the USISS scheme during the 2013/14, 2014/15, and 2015/16 influenza seasons. Of these 227 were eligible for inclusion in the study, and the remaining 170 cases were excluded (Figure 1). These cases were excluded for reasons including cases not being in a target group for vaccination in the study seasons (*n* = 106), non‐English residents (*n* = 1), missing vaccination status (*n* = 25), more than 7 days between date of onset and swab date (*n* = 6), received IIV (*n* = 18), vaccination took place within 14 days of illness onset (*n* = 10), missing date of vaccination (*n* = 3), and influenza subtype recorded as other (*n* = 1) (Figure 1).

Of the 227 included cases, 69.6% occurred in the 2015/16 influenza season (*n* = 157), 21.6% in the 2014/15 season (*n* = 49), and the remaining 8.8% (*n* = 20) in the 2013/14 season (Table 2). 50.7% were due to influenza A(H1N1pdm09) (*n* = 115), 15.4% due to influenza A(H3N2) (*n* = 33), 23.8% due to influenza B (*n* = 54), and 10.1% due to influenza A unknown subtype (*n* = 23).

**TABLE 2:** Characteristics of influenza cases (*n* = 227)

	Number (column %)	Number vaccinated (row %)	Number unvaccinated (row %)
Season			
2013/14	20 (8.8)	6 (30.0)	14 (70.0)
2014/15	49 (21.6)	11 (22.4)	38 (77.6)
2015/16	158 (69.6)	38 (24.1)	120 (75.9)
Age group			
2 years	79 (34.8)	18 (22.8)	61 (77.2)
3 years	65 (28.6)	17 (26.2)	48 (73.8)
4 years	58 (25.6)	14 (24.1)	44 (75.9)
Year 1 (aged 5 rising to 6 years)	14 (6.2)	1 (7.1)	13 (92.9)
Year 2 (aged 6 rising to 7 years)	11 (4.8)	5 (45.5)	6 (54.5)
Sex			
Male	135 (59.5)	31 (23.0)	104 (77.0)
Female	92 (40.5)	24 (26.1)	68 (73.9)
PHE region			
North of England	109 (48.0)	26 (23.9)	83 (76.1)
South of England	48 (21.1)	12 (25.0)	36 (75.0)
Midlands and East of England	37 (16.3)	8 (21.6)	29 (78.4)
London	31 (13.7)	8 (25.8)	23 (74.2)
Unknown	2 (0.9)	1 (50.0)	1 (50.0)

Abbreviation: PHE, Public Health England.

Fifty‐five of the 227 cases included (24.2%) were vaccinated. Of these, all cases were vaccinated with the LAIV (*n* = 53), apart from two cases with vaccine type unknown (*n* = 2).

### 3.2 Description of reference population

The population vaccine coverage for England for the population groups eligible for vaccination in the study seasons are shown in Table 3. Uptake was generally higher in all population groups compared with the PCV (Table 3).

**TABLE 3:** Vaccination status in cases reported to UK Severe Influenza Surveillance System (USISS) compared with national cumulative influenza vaccine uptake and crude and adjusted influenza vaccine effectiveness by age group and season, England

	Percentage of cases vaccinated (PCV)^a^	Percentage of reference population vaccinated (PPV)^b^	Crude VE (95% CI)	Adjusted VE^c^ (95% CI)
2013/14				
2–3 years	30.0 (6/20)	41.1 (594 610/447 303)	38.5 (−70.2, 80.6)	50.5 (−39.0, 82.3)
2014/15				
2–4 years	22.4 (11/49)	37.6 (828 663/204 408)	51.9 (4.1, 77.8)	43.3 (−12.1, 71.3)
2015/16				
2–4 years	24.1 (32/133)	34.4 (728 066/119 123)	39.5 (9.1, 60.7)	49.4 (22.8, 66.9)
Year 1 + 2 combined (5–6‐year‐olds)	24.0 (6/25)	53.6 (716 928/336 603)	46.1 (25.2, 61.8)	62.6 (2.6, 85.6)

Abbreviations: CI, confidence interval; PPV, proportion of the population vaccinated; VE, vaccine effectiveness.

^a^ At least 14 days prior to symptom onset.

^b^ % vaccinated by January 31.

^c^ Adjusted VE matched by CCG/local authority, age in years, week/month of infection.

### 3.3 VE estimates

The crude and adjusted VE estimates for preventing influenza‐hospitalized cases in children by season and age group, for all influenza types, are shown in Tables 3 and 4.

**TABLE 4:** Crude and adjusted influenza vaccine effectiveness estimates overall, by age group and influenza type, 2013/14–2015/16

	Crude VE (95% CI)	Adjusted VE^a^ (95% CI)
Overall (2013/14–2015/16)	52.7 (35.6, 65.8)	50.1 (31.2, 63.8)
By season (main circulating strain)		
2013/14 (influenza A/H1N1pdm09)	38.5 (−70.2, 80.6)	50.5 (−39.0, 82.3)
2014/15 (influenza A/H3N2 & B)	51.9 (4.1, 77.8)	43.3 (−12.1, 71.3)
2015/16 (influenza A/H1N1pdm09 & B)	55.9 (36.1, 70.2)	52.0 (29.2, 67.5)
By age group		
2–4‐year‐olds	46.1 (25.2, 61.8)	48.1 (27.2, 63.1)
Year 1 + 2 combined (5–6‐year‐olds)	72.7 (28.9, 91.1)	62.6 (2.6, 85.6)
By flu type		
Influenza A (all subtypes)	49.6 (28.5, 65.0)	45.0 (21.3, 61.6)
Influenza A/H1N1pdm09	47.8 (20.1, 66.6)	44.0 (13.8, 63.5)
Influenza A/H3N2	48.8 (−12.7, 78.9)	49.7 (−17.4, 78.5)
Influenza A unknown subtype	58.9 (−14.7, 88.1)	43.8 (−54.5, 79.5)
Influenza B	62.2 (25.4, 82.4)	65.0 (27.3, 83.1)

Abbreviations: CI, confidence interval; VE, vaccine effectiveness.

^a^ Adjusted VE by CCG/local authority, age in years (where appropriate), week/month of infection.

The overall adjusted LAIV VE for all influenza types pooled over the three influenza seasons and all age groups was 50.1% (95% confidence interval [CI] 31.2, 63.8) (Table 4). By age, there was good evidence of protection against hospitalization from LAIV in both the pre‐school age group (2–4‐year‐olds) (48.1, 95% CI 27.2, 63.1, *p* < 0.000) and in school aged children (Years 1 and 2) (62.6%, 95% CI 2.6, 85.6, *p* = 0.044) over the three influenza seasons. Results after stratifying by influenza type gave an adjusted VE in children (pre‐school and Years 1 and 2) of 44.0% against influenza A/H1N1pdm09, 49.7% against influenza A/H3N2, 43.8% against influenza unknown subtype, and 65.0% against influenza B (Table 4).

Adjusted VE estimates were varied by age group and season. The LAIV vaccine showed good protection against hospitalization in 2015/16 in children in both the pre‐school (49.4%, 95% CI 22.8, 66.9) and school cohort (62.6, 95% CI 2.6, 85.6) (Table 3). Good point estimates of protection were also seen for 2–3‐year‐olds in 2013/14 (50.5, 95% CI −39.0, 82.3) and 2–4‐year‐olds in 2014/15 (43.3, 95% CI −12.1, 71.3), although with wide CIs overlapping zero (Table 3).

A high proportion of 2‐ to 4‐year‐old cases had missing information on risk group status (66.8%). A slightly greater proportion of unvaccinated cases had missing information on risk group status compared with vaccinated cases (69.9% compared with 57.1%). When stratified by risk group, the adjusted VE for those with a risk factor was −8.0% (95% CI −117.6, 46.4) and for those with no risk factor, VE was 50.7% (95% CI −22.5, 80.2).

In a further subgroup analysis restricted to 2–4‐year‐olds on whom risk factor status was known, VE estimates were also adjusted for presence/absence of a risk factor (Table 5). The combined VE for 2–4‐year‐olds with known risk factor after adjusting for risk factor, as well as geography, week, and age was 44.2%. (95% CI 3.3, 67.8) compared with 48.1% (95% CI 27.2, 63.1) without adjusting for underlying risk factor (Table 5).

**TABLE 5:** Vaccine effectiveness estimates adjusted for risk group status for children aged 2–4 years, and sensitivity analysis

Age group	Adjusted VE (all cases, no adjustment for risk group)	Adjusted VE* (restricted to cases with known risk group status) (95% CI)	Adjusted VE (cases with unknown risk group status assumed to have risk factor) (95% CI)	Adjusted VE (cases with unknown risk group status assumed to have no risk factor) (95% CI)
2–4‐year‐olds	48.1 (27.2, 63.1)	44.2 (3.3, 67.8)	69.9 (57.6, 78.6)	57.6 (34.1, 72.7)

Abbreviations: CI, confidence interval; VE, vaccine effectiveness.

* Adjusted for risk factor in addition to CCG, week, and age.

In a sensitivity analysis, cases where risk group status was unknown were assumed to have a risk factor, and the appropriate vaccine uptake for presence of a risk group was used. The adjusted VE in this instance was 69.9% (95% CI 57.6, 78.6) for all 2–4‐year‐olds (Table 5). Alternatively, the adjusted VE when cases with missing risk group status were assumed to have no risk factor was 57.6% (95% CI 34.1, 72.7) in all 2–4‐year‐olds (Table 5).

## 4 CONCLUSIONS

In this study, the screening method was used to evaluate the effectiveness of influenza vaccination in preventing influenza‐associated hospitalizations in children during the 2013/14–2015/16 influenza seasons in England: the first three seasons following the introduction of the childhood influenza vaccination program. Overall adjusted LAIV effectiveness against hospitalization in children over the three seasons was 50.1%.

Using the screening method, vaccination coverage for confirmed influenza hospitalizations was collected through a severe disease surveillance system and compared with population vaccination coverage obtained through a national vaccine uptake monitoring system. Overall LAIV vaccine uptake among the hospitalized cases was 24.2%. Adjusted influenza VE against hospitalization showed good evidence of protection against hospitalization in both pre‐school children (48.1%) and school‐aged children (62.6%) over the three seasons. By season, good protection was seen in the 2015/16 season, when influenza A/H1N1pdm09 and influenza B dominated and the A/Bolivia strain was the vaccine strain.^31^ This provides reassurance that the vaccine continued to provide protection against severe disease over these seasons.

VE was good against influenza B (65.0%) and influenza A/H1N1pdm09 (44.0%), although non‐significant results were seen against influenza A/H3N2 subtype, the dominant circulating subtype in 2014/15. This was likely due to mismatch between the strains included in the vaccine and the circulating influenza A/H3N2 strain in the 2014/15 season.^32^

Recent studies have found mixed results regarding inclusion of the presence/absence of underlying medical risk factors as a confounder of VE estimates.^19^, ^23^, ^32^, ^33^ It is, theoretically, a possible important confounder of the vaccination–influenza effect, because the presence of certain medical conditions may increase a person's risk of severe influenza, as well as being an eligibility criterion for free vaccination in certain countries.^34^

For the reference population uptake, risk factor status was only available for the 2‐ to 4‐year‐old group, although it was missing for 66.8% of 2‐ to 4‐year‐old hospitalized cases. When restricting the analysis to 2–4‐year‐olds with known risk factor status, the overall adjusted VE for 2–4‐year‐olds reduced slightly from 48.1% to 44.2%. To assess the robustness of the conclusions, a sensitivity analysis was performed to explore the effects of missing risk factor information further. Assuming all those to have an underlying risk factor or all assumed not to have an underlying risk factor made only a small difference to the VE estimate (69.9% compared to 57.6%) for 2–4‐year‐olds.

Our findings are consistent with a similar study that used the screening method to assess VE against hospitalization in 2–6‐year‐olds in England in the 2015/16 influenza season.^24^ The overall adjusted VE in this study was similar to our estimate for 2015/16 (54.5% compared with 52.0%) with overlapping CIs. Our 2015/16 season findings are also consistent, although slightly higher, with a test negative design (TND) study that also assessed LAIV VE against hospitalization in children in 2015/16 in England.^23^ This TND study found an overall adjusted VE against hospitalization in children aged 2–16 years of 41.9%.^23^ In this study, risk factor was controlled for along with age, sex, risk group, region, and sample month, as well as index of multiple deprivation and ethnicity.

There are several strengths and limitations to our study. This study utilised an established severe disease surveillance system to identify hospitalized cases that allowed key variables to be collected on the cases. In addition, a write back to the case's GPs was conducted and provided a rapid and cheap method of obtaining vaccine uptake status on the cases while achieving high levels of completion of vaccine information. This study also benefitted from an existing, national vaccine uptake monitoring system, Immform, to provide population vaccine uptake figures. A strength of this national uptake data is that it is available by some of the key potential confounding variables in examining associations between vaccination and hospitalization for influenza.

One of the main limitations of the screening method is that VE estimates may be biased if cases arise from a population that differs from the population used to determine vaccine coverage rates. We have attempted to address this by comparing the vaccine coverage among cases to the uptake in the general population to the same age in the local area where cases lived. Where possible we also compared uptake by risk group status, however, this was poorly completed on cases and not available for all population vaccination groups, specifically the school‐based groups. A second potential limitation of the screening method is the inability to adjust for important confounders, particularly due to lack of detail on key confounders on the population coverage. In this study, we were able to adjust for age, time of infection, place of residence and, for some age groups, risk group status; however, vaccine type was not available, so it was not possible to assess if VE differed by vaccine type. Another limitation of this study specifically was that illness onset dates were missing for a proportion of cases; however, we were able to use alternative dates as a proxy for onset date. Okoli et al. suggest that accurate reporting of symptom onset is crucial for TND studies and, specifically, that symptom onset should be restricted to 7 days or less, which is likely to also be applicable to the screening method.^35^

In summary, we have found that vaccination with LAIV provided good protection against laboratory‐confirmed influenza infection resulting in hospitalization in England over the 2013–2015 influenza seasons. The screening method provides a rapid and cheap method to estimate influenza vaccination effectiveness overall and by influenza subtype. The study highlights the importance of having clinical risk factor information available for both cases and the reference population. Optimizing the completeness of data such as swab dates, vaccination status and dates, and risk factor status could improve the validity of VE estimates using this method. The results of this study of VE against hospitalization with influenza support the roll‐out of the childhood influenza vaccination program in the United Kingdom. These findings are of importance as this current influenza season sees the further expansion of the program to secondary school children in England and of relevance to other countries considering introducing childhood influenza vaccination programs.

## FUNDING INFORMATION

This paper receives no funding.

## AUTHOR CONTRIBUTIONS

**Nicki Boddington:**Data curation; formal analysis; methodology; project administration. **Punam Mangtani:** Methodology; supervision. **Hongxin Zhao:** Data curation; project administration. **Neville Verlander:** Formal analysis. **Joanna Ellis:** Data curation. **Nick Andrews:** Formal analysis. **Richard Pebody:** Conceptualization; data curation; methodology; supervision.

### PEER REVIEW

The peer review history for this article is available at https://publons.com/publon/10.1111/irv.12990.

## Data Availability

The data that support the findings of this study are available from the corresponding author upon reasonable request.
